# Origin of measles virus: divergence from rinderpest virus between the 11^th ^and 12^th ^centuries

**DOI:** 10.1186/1743-422X-7-52

**Published:** 2010-03-04

**Authors:** Yuki Furuse, Akira Suzuki, Hitoshi Oshitani

**Affiliations:** 1 Department of Virology, Tohoku University Graduate School of Medicine, Sendai city, Japan

## Abstract

Measles, caused by measles virus (MeV), is a common infection in children. MeV is a member of the genus *Morbillivirus *and is most closely related to rinderpest virus (RPV), which is a pathogen of cattle. MeV is thought to have evolved in an environment where cattle and humans lived in close proximity. Understanding the evolutionary history of MeV could answer questions related to divergence times of MeV and RPV.

We investigated divergence times using relaxed clock Bayesian phylogenetics. Our estimates reveal that MeV had an evolutionary rate of 6.0 - 6.5 × 10^-4 ^substitutions/site/year. It was concluded that the divergence time of the most recent common ancestor of current MeV was the early 20^th ^century. And, divergence between MeV and RPV occurred around the 11^th ^to 12^th ^centuries. The result was unexpected because emergence of MeV was previously considered to have occurred in the prehistoric age.

MeV may have originated from virus of non-human species and caused emerging infectious diseases around the 11^th ^to 12^th ^centuries. In such cases, investigating measles would give important information about the course of emerging infectious diseases.

## Findings

Measles is a common infection in children and is spread by the respiratory route. It is characterized by a prodromal illness of fever, coryza, cough, and conjunctivitis followed by appearance of a generalized maculopapular rash. Measles virus (MeV) infects approximately 30 million people annually, with a mortality of 197,000, mainly in developing countries [1]. In the prevaccine era, more than 90% of 15-year-old children had a history of measles [2]. Measles remains a major cause of mortality in children, particularly in areas with inadequate vaccination and medical care.

MeV infection can confer lifelong immunity [3,4], and there is no animal reservoir or evidence of latent or common persistent infection except for subacute sclerosing panencephalitis (SSPE). Therefore, maintenance of MeV in a population requires constant supply of susceptible individuals. If the population is too small to establish continuous transmission, the virus can be eliminated [5]. Mathematical analyses have shown that a naïve population of 250,000-500,000 is needed to maintain MeV [6-8]. This is approximately the population of the earliest urban civilizations in ancient Middle Eastern river valleys around 3000-2500 BCE [6,9,10]. Historically, the first scientific description of measles-like syndrome was provided by Abu Becr, known as Rhazes, in the 9^th ^century. However, small pox was accurately described by Galen in the 2^nd ^second century whereas measles was not. Epidemics identified as measles were recorded in the 11^th ^and 12^th ^centuries [9-11].

MeV is a member of the genus *Morbillivirus*, which belongs to the family *Paramyxoviridae *[12]. In addition to MeV, *Morbillivirus *includes dolphin and porpoise morbillivirus, canine distemper virus, phocid distemper virus, peste des petits ruminants virus, and rinderpest virus (RPV) [12,13]. Genetically and antigenetically, MeV is most closely related to RPV, which is a pathogen of cattle [12,14]. MeV is assumed to have evolved in an environment where cattle and humans lived in close proximity [11]. MeV probably evolved after commencement of livestock farming in the early centers of civilization in the Middle East. The speculation accords with mathematical analyses as mentioned above [6,9,10].

Molecular clock analysis can estimate the age of ancestors in evolutionary history by phylogenetic patterns [15,16]. The basic approach to estimating molecular dates is to measure the genetic distance between species and use a calibration rate (the number of genetic changes expected per unit time) to convert the genetic distance to time. Pomeroy et al. showed that "Time to the Most Recent Common Ancestor" (TMRCA: the age of the sampled genetic diversity) of the current MeV circulating worldwide is recent, i.e., within the last century (around 1943) [17]. Nevertheless, the time when MeV was introduced to human populations has not been investigated until date. In the present study, we performed molecular clock analysis on MeV to determine the time of divergence from RPV, suggesting the evolutionary path of the virus.

MeV sequences were downloaded from GenBank and aligned using ClustalW. Additional file 1 includes a list of accession numbers for sequences used in this study. Sequences of the hemagglutinin (H) and nucleocapsid (N) genes collected worldwide between 1954 and 2009 were used. The H and N genes were selected for analyses since their sequences are registered commonly. Sequences associated with the persistent disease manifestation SSPE were removed because these were expected to exhibit different evolutionary dynamics [18]. To avoid weighting specific outbreaks, we also excluded sequences that had been collected at the same time and place and that were genetically similar to each other. Consequently, the final data sets comprised 149 taxa with an alignment length of 1830 bp for the H gene and 66 taxa with an alignment length of 1578 bp for the N gene.

To determine the divergence time between MeV and RPV, sequences of peste des petits ruminants virus [GenBank: FJ750560 and FJ750563] were used to define the root of divergence between MeV and RPV.

The rates of nucleotide substitutions per site and TMRCA were estimated using the Bayesian Markov chain Monte Carlo (MCMC) method available in the BEAST package [19,20]. This method analyzes the distribution of branch lengths among viruses isolated at different times (year of collection) among millions of sampled trees. For each data set, the best-fit model of nucleotide substitution was determined using MODELTEST [21] in HyPhy [22]. All models were compared using Akaike's Information Criterion. For both the H and N genes, the favored models were closely related to the most general GTR + Gamma + Inv model. Statistical uncertainty in parameter values across the sampled trees was expressed as 95% highest probability density (HPD) values. Runs were carried out with chain lengths of 100 million and the assumption of an 'exponential population growth' using a 'relaxed (uncorrelated lognormal) molecular clocks' [23]. All other parameters were optimized during the burn-in period. The output from BEAST was analyzed using the program TRACER http://beast.bio.ed.ac.uk/Tracer. BEAST analysis was also used to deduce the maximum a posteriori (MAP) tree for each data set, in which tip times correspond to the year of sampling.

**Figure 1:**
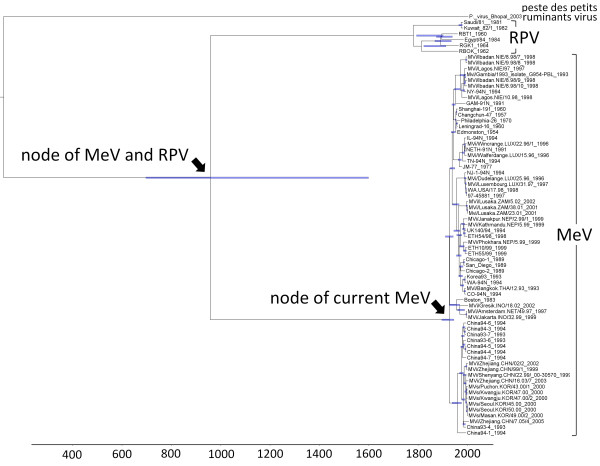
**Bayesian estimates of divergence time**. Maximum a posteriori (MAP) tree of the N gene. Tip times reflect the year of sampling. Internal nodes have error bars of 95% credible intervals on their date.

The Bayesian approach assumed varied rates by branch. Using the Bayesian estimate, our analysis derived a mean evolutionary rate of 6.02 × 10^-4 ^substitutions/site/year for the N gene and 6.44 × 10^-4 ^substitutions/site/year for the H gene (Table 1). Based on this approach by analyses for the N gene, 1921 was estimated to be the TMRCA of the current MeV (Figure 1). Date of divergence between MeV and RPV was 1171. Analyses for the H gene yielded similar results; the TMRCA of the current MeV was 1916. 1074 was estimated to be the date of divergence between MeV and RPV.

**Table 1:** Analysis profiles

Gene	Evolutionary rate, substitutions/site/year (95% HPD)	TMRCA of the current MeV (95% HPD)	Time of divergence between MeV and RPV (95% HPD)
N	6.02 × 10^-4 ^(3.62, 8.76)	1921 (1895, 1945)	1171 (678, 1612)
H	6.44 × 10^-4 ^(3.65, 9.25)	1916 (1889, 1944)	1074 (437, 1576)

HPD, Highest probability density

Our results indicate that divergence of MeV from RPV occurred around the 11th to 12th centuries. The population size at that time was sufficient for maintaining MeV. However, this result was unexpected because emergence of MeV was previously considered to have occurred in the prehistoric age [6,7,9,10]. Estimation errors seem unlikely since Bayesian approach yielded results which are compatible with other reports. In general, substitution rates between 10^-3 ^and 10^-4 ^substitutions/site/year have been previously estimated for RNA viruses including MeV [17,24,25]. Pomeroy et al. also found that the date of divergence of the current MeV was within the last century [17].

In the prevaccine era, over 90 percent of children is infected with MeV by age 15 [2]. Nevertheless, measles has been rarely described earlier. An increasing number of descriptions of measles in the 11^th ^and 12^th ^centuries may reflect the emergence of MeV in human populations at that time [9-11]. Linguistic evidence suggests that the disease was recognized before the Germanic migrations but after the fragmentation of the Roman Empire, i.e., between 5^th ^and 7^th ^centuries [10,11]. This age is still within 95% credible intervals of our results. Alternatively, a common ancestor of MeV and RPV may have caused zoonosis in the past; the archaeovirus can infect both humans and cattle. Even if the earliest urban civilizations in ancient Middle Eastern river valleys (around 3000 to 2500 BCE) were infected by an ancestor of the current MeV, the virus probably had different characteristics from the current MeV.

Emerging infectious diseases have recently caused significant morbidity and mortality. Many diseases are caused by viruses originating in non-human species [26]: HIV from non-human primates [27]; SARS coronavirus from bats [28]; and the pandemic strain of influenza virus in 2009 from swine [29]. MeV may have originated from non-human species and caused emerging infectious diseases around the 11^th ^to 12^th ^centuries. In such cases, investigating measles would give important information about the course of emerging infectious diseases after their introduction into the human population, from evolutionary and epidemiological perspectives.

## List of Abbreviation

MeV: measles virus; RPV: rinderpest virus; TMRCA: Time to the Most Recent Common Ancestor; H: hemagglutinin; N: nucleocapsid.

## Competing interests

The authors declare that they have no competing interests.

## Authors' contributions

YF carried out all analyses and drafted the manuscript. AS and HO participated in the design of the study and helped to draft the manuscript. All authors have read and approved the final manuscript.

## Supplementary Material

Additional file 1 **List of accession numbers**. The file contains list of accession numbers of sequencing data we analyzed. Click here for file
